# Rejection in romantic relationships: Does rejection sensitivity modulate emotional responses to perceptions of negative interactions?

**DOI:** 10.1186/s40359-024-01864-w

**Published:** 2024-06-25

**Authors:** Marianne Richter, Georgia Kouri, Nathalie Meuwly, Dominik Schoebi

**Affiliations:** 1 Department of Psychology, University of Fribourg, Rue de Faucigny 2, Fribourg, 1700 Switzerland; 2 Institute of Psychology, University of Lausanne, Lausanne, 1015 Switzerland

**Keywords:** Rejection sensitivity, Intimate relationships, Emotions, Rejection, Perceptions

## Abstract

**Background:**

Rejection is a highly stressful experience and individuals tend to avoid it whenever possible. In intimate relationships, experiences of rejection can shape the interaction dynamics between partners. Highly rejection sensitive people fear that their romantic partner will reject them and they overreact to any ambiguous cues that might indicate rejection. Furthermore, because they focus on the threat of rejection, they may have difficulty disengaging from rejection-related emotions, persevere in a rejection-focused state and have a reduced capacity to regulate their emotions. The prolonged experience of strong negative emotions, together with maladaptive attempts to respond to rejection, may undermine key relationship maintenance processes that contribute to relationship functioning and lead to negative reciprocity in interactions. The goal of the present study was to shed light on how individuals experience rejection-related emotions and determine whether, following perceptions of negative interactions, rejection sensitivity was associated with stronger negative responses and less efficient downregulation of negative emotions. In addition, we examined whether dyadic patterns of rejection sensitivity were associated with negative emotion dynamics following perceptions of negative interactions.

**Methods:**

The participants (*N* = 298) were couples experiencing the transition to parenthood. A multilevel modelling approach was used to assess the associations between rejection sensitivity, perceptions of negative interactions and emotional states. The analyses included repeated daily reports for both rejection and emotions.

**Results:**

The results suggest that rejection sensitive individuals do not report higher negative emotions when they perceive negative interactions. Moreover, rejection sensitive men and women did not remain longer in a negative emotional state after they perceived negative interactions with their partner. Finally, when both men and women partners reported higher levels of rejection sensitivity, neither reported having higher negative emotions after experiencing negative interaction perceptions.

**Conclusions:**

Our findings provide further insights into emotional dynamics and rejection sensitivity in romantic relationships. Our results do not provide evidence for a link between rejection sensitivity and higher negative emotions or slower recovery after reports of negative interactions. If individuals suppress their emotions, they may not benefit from regulation with their partner and instead may protect themselves over their relationships. However, in this context, rejection sensitivity might also not constitute a strong predictor of daily emotion fluctuations, but other variables– such as relationship satisfaction – might. Future research may investigate emotional responses in a sample with higher levels of rejection sensitivity and use more diverse measures of perceptions of negative interactions.

For better or for worse, intimate relationships affect us emotionally [1]. Social rejection is also a painful interpersonal experience [2, 3] and feeling rejected by an intimate partner may be particularly hurtful because people reveal their most vulnerable side in their intimate relationships [4]. It may not be surprising, therefore, that critical interpersonal situations with a perceived risk of rejection trigger anxiety and negative emotions. However, people likely differ in the extent to which they suffer emotionally and recover from the pain of rejection [2, 5]. Rejection sensitive individuals are thought to be more sensitive and vigilant to rejection than other individuals [6]. On the one hand, this may contribute to their anxiety in interpersonal situations with their partner, they may perceive rejection more readily [6] and show stronger and more lasting emotional responses to negative or ambiguous interactions. On the other hand, as a self-protective strategy, these individuals may become less emotionally involved with their partners, which limits the risk of acute rejection experiences [7]. Identification of such tendencies is important, as they may undermine lasting closeness and intimacy [8, 9]. The current research examined the associations between rejection sensitivity and emotional responses after potentially hurtful interpersonal situations.

## Perceptions of rejection in romantic relationships

Rejection experiences can be highly subjective and involve the interpretation of social cues. They may therefore arise in diverse interpersonal situations, such as perceiving a lack of support when support is expected, or experiencing unresponsive behaviours or disinterest from the partner [10]. For instance, self-report perceptions that the partner acted in a hurtful way, was critical or unpleasant, have been used as proxies for rejection-related events [10, 11]. Therefore, conflicts and tense interpersonal situations, as well as other situations where loyalty, support, approval, or validation are expected from a partner, bear the potential to make an individual feel threatened and rejected [2, 11]. Because experiencing unconditional acceptance from a close partner is a basis for feeling validated, supported and understood [12], rejection cues are hurtful and undermine intimacy [4]. People are therefore motivated to try and avoid or prevent such experiences [13, 14] and are likely to show strong affective reactions if they occur [15, 16].

### Rejection sensitivity and emotional responses to perceived rejection

Because perceived rejection jeopardises an individual’s sense of acceptance, it triggers negative emotions and motivates regulatory behaviours to help them cope with the threat and restore acceptance [15, 17, 18]. Indeed, experimental manipulations of rejection cause individuals to experience more intense sadness and hurt feelings [18]. These emotional responses accompany negative behavioural responses, which may prolong negative interactions and interfere with the downregulation of negativity. For example, situations that elicit jealousy are perceived as rejecting and not only foster anger and fear but can also prompt aggressive behaviours [19–21].

In intimate relationships, rejection sensitive individuals tend to anxiously expect rejection from their partner and therefore readily perceive ambiguous cues as rejection [6]. High levels of rejection sensitivity have been linked to higher levels of emotional or behavioural dysregulation, and insecure attachment [6, 22, 23]. Rejection sensitive individuals often react strongly to ambiguous situations by providing diminished emotional support or they respond with maladaptive behaviours, such as controlling and self-silencing behaviours [6, 24, 25]. They may have learnt that seeking support or acceptance from a significant other may lead to rejection [6]; hence, anxious expectations of rejection foster hypervigilance to rejection-related cues. As a result, rather than being responsive and supportive, rejection sensitive individuals are prone to show defensive and self-protective behaviours in critical relational situations [6]. In turn, their partners may reciprocate their negative behaviours, which ultimately results in prolonged interpersonal distress [7].

The disposition to be sensitive to negative cues and respond to them with negative emotions and/or defensive or hostile behaviour is likely to increase difficulties with emotion regulation and adjustment in intimate relationships [1, 26]. Consequently, negative emotional states may persist over time. For example, rejection sensitive students in committed relationships show more emotional distance from their partner, and in turn, are more dissatisfied in their close relationships [6, 27]. Rejection sensitivity is also associated with increased reciprocation of the partner’s behaviours and attitudes. While rejection sensitive individuals are warm in response to their partner’s positive affect, they are distant and cold toward a partner’s negative affect. Importantly, when a significant other is distressed, rejection sensitive individuals fail to respond with warmth [28]. In a similar vein, anxious and avoidant attachment orientations are associated with reactions to rejection, although in different ways; whereas anxious individuals show more personal distress and guilt in response to rejection, avoidant individuals tend to respond with greater hostility or emotional suppression [11, 29]. It is important to consider these contrasting differences when individuals are faced with rejection or are more sensitive to threatening interactions with their partners.

It is also possible that rejection sensitive individuals try to prevent negative emotional states that result from expected rejection. For example, they may avoid intimacy and closeness situations because they involve the risk of feeling rejected [7] and the negative affective experiences associated with rejection. Hence, keeping emotional distance from the partner and disconnecting from rejection-related emotions may appear as a viable strategy to reduce the experience of rejection [25, 27]. Rejection sensitive individuals, like those with an avoidant attachment style or individuals with low self-esteem, may prefer to maintain a sense of safety by reducing their closeness to significant others [4, 7, 20]. The risk regulation model [4, 30, 31] offers an interesting framework to better understand partner interactions. The model explains how in interdependent relationships, individuals must take risks and show themselves as vulnerable to fulfil their need for connectedness and closeness to their partners. Accordingly, if an individual evaluates a situation as safe and their partner as accepting and responsive to their needs, they tend to choose self-disclosure over distancing. However, if they doubt that their partner will be responsive, they prefer self-protective behaviours over self-disclosure and connection [32] If individuals are prone to perceive rejection, they are likely to behave in self-protecting ways even in situations that harbour no objective threat [25]. As a result, they are likely to miss opportunities for building and maintaining intimate bonds with their partner and instead worry about rejection. This may further contribute to their emotional instability and impaired interpersonal adjustment [1, 33], potentially prolonging their negative emotional response to rejection.

Taken together, negative emotional and behavioural responses to perceived rejection are likely to trigger further cues of rejection from the partner [28, 34]. These responses may interfere with the downregulation of negative emotions and interpersonal adjustment [5, 6]. Therefore, rejection sensitive individuals may focus more persistently on and perceive threat cues from their partner, thus remaining in a negative emotional state for longer.

### Dyadic patterns of rejection sensitivity and negative emotions in interpersonal interactions

Rejection sensitive people may find it difficult to disengage from rejection-related emotions and thoughts in the face of tense or ambiguous interactions with their partner. They may respond in maladaptive ways to the rejection cues they perceive from their partner, which fosters continued distressing interactions [5]. Arguably, if we assume that rejection sensitive individuals show stronger emotional reactivity to negative or ambiguous cues from their partner, then having a partner who tends to show accommodating behaviour will dampen further negative responses. However, having a rejection sensitive partner who is susceptible to showing negative reactions to negative or ambiguous cues will amplify the negativity. Thus, if both partners are prone to perceiving rejection and reacting negatively to such perceptions [5, 34], a negative feedback loop may occur, with both partners reciprocating each other’s negative affective responses. Similar effects of dyadic patterns between partners have been examined regarding the associations between attachment orientation and communication [35] and between attachment and coregulation of affect [36]. Specifically, a study of avoidance-oriented individuals showed that they were more likely to communicate in a negative way if their partner also displayed negative communication behaviours [37]. To our knowledge, no studies have investigated the effects of dyadic patterns of rejection sensitivity, even though it seems plausible that a partner’s response tendencies are relevant to the outcomes of interactions. Investigating dyadic effects may therefore contribute to our understanding of the role of rejection sensitivity in intimate interaction dynamics.

Therefore, along with a reduced capacity to regulate emotions [13], we would expect that elevated rejection sensitivity in both partners would give rise to increased negative reciprocal dynamics in distressing interactions and contribute to more prolonged negativity. In contrast, in couples where only one partner shows elevated rejection sensitivity, these dynamics and emotional responses might be still present but less pronounced, because the other partner may buffer the negativity during dyadic interactions.

### The current study

In the current study, we examined emotional responses to perceptions of negative interactions, including rejection or disregard from a romantic partner. We tested the individual and dyadic effects of rejection sensitivity on emotional change associated with or subsequent to negative interactions. Specifically, we expected that, when perceiving negative interactions, rejection sensitive individuals would report stronger increases in negative emotions than individuals low in rejection sensitivity (Hypothesis 1; H1). Furthermore, we expected that rejection sensitive individuals would show a less rapid downregulation of negative emotions (Hypothesis 2; H2).^1^ Finally, we expected that dyadic patterns (combinations) of rejection sensitivity would predict increased negative emotions following perceptions of negative interactions, above and beyond the rejection sensitivity of the two partners (Hypothesis 3; H3). We expected that mutually high levels of rejection sensitivity in both partners would be associated with stronger negative emotions (Hypothesis 3a; H3a), and a reduced downregulation of negative emotions over time (Hypothesis 3b; H3b), than in dyadic patterns of one partner or none of the partners being high in rejection sensitivity.^2^ Because rejection sensitivity has been associated with marital dissatisfaction in both rejection sensitive individuals and their partners [6] we included relationship satisfaction as a control variable. To test our hypotheses, we used data from the first measurement in a project on couples’ transition to parenthood, collected before the birth of their child.

The present study was preregistered (during data collection) on the open science platform osf.io (https://osf.io/wyz4r*).* To assess emotion dynamics and rejection, we used an ecological momentary assessment approach in that the participants reported their daily experiences and emotional states four times per day over seven consecutive days. These data allowed us to model within-person variability of emotional states and trends of within-person emotional change over time, as associated with negative interpersonal interactions and rejection sensitivity, using a multilevel modelling approach.

## Materials and methods

### Participants

Participating couples were recruited during the second or third trimester of their first pregnancy, and data collection took place in the second or third trimester of pregnancy (further measurements, which were not included in this current study, were taken at six, 12 and 18 months after birth). Recruitment began in March 2019 and ended in February 2022. The inclusion criteria included being in a mixed-gender relationship, being fluent in one of the study languages (German or French), being over 18 years old, living in a shared household with their partner, and expecting their first child. Each couple was compensated with the equivalent of approximately 900 USD for participation in all parts of the study.

The participants were recruited via flyers through midwives, gynaecologists, birth centres and prenatal courses, as well as via social media and word-of-mouth. Additionally, posters were distributed in universities, pharmacies, supermarkets, and hospitals. Potential participants could contact the study team via email or phone to obtain more information about the study. They were provided with a detailed information sheet and had the opportunity to ask questions and further discuss their potential participation in the study with a researcher on the phone. Both partners were required to provide consent to participate in the study.

By December 2021, 149 couples (*N* = 298 participants) had completed the first part of the couples’ transition to parenthood study, and their data were used for the current study. The average age was 31.55 years (*SD* = 3.67) for women and 33.20 years (*SD* = 4.06) for men. At the time of recruitment, couples had been in a relationship for an average of 6.73 years (*SD* = 3.01). The participants reported a relatively high level of education, with 65.6% of the sample holding a university degree, 11.9% had another type of advanced education/training, 10.3% had completed an apprenticeship, 5.6% were undergraduate students, 5% had completed high school, and 1.7% had a secondary school degree. At the time of data collection, 70.9% of the sample were employed, 9.6% were executive employees or in a managerial position, 10.9% were self-employed, and 8.6% were not working. The median individual net income was the equivalent of approximately 5600 USD.

### Procedure

All the couples completed the first set of assessments in the second or third trimester of pregnancy. All the participants were informed about the nature of the procedure and both partners provided informed consent before data collection began. The data used in the present study were collected as part of an online questionnaire completed after enrolment in the study, and a seven-day smartphone-based momentary assessment that began after the participants completed the online questionnaire. The online questionnaire included questions about the participants’ relationship and their mental health and well-being.

Ecological momentary assessments were prompted four times per day (8:00 a.m., 12:00 p.m., 6:00 p.m. and 9:30 p.m.) over seven consecutive days. Both partners were prompted to complete the assessment at the same time. Adherence was good (*M* = 26.7 reports per week out of a maximum of 28 reports; 4.52% missing data). The momentary assessment included questions about the participant’s daily interactions with their partner, their own and their partner’s affect, interpersonal behaviours, stress, perceptions of negative interactions, intimacy, and relationship satisfaction. Other assessments in the main study included a mental health diagnostic interview, home visits with interaction tasks, and physiological measures during the home visits and on three consecutive days of their daily lives (cortisol and heart rate frequency); these data were not part of the current study. The project obtained approval from the ethics review board of the regional government.

### Measures

#### Adult rejection sensitivity

Rejection sensitivity was measured using the Adult Rejection Sensitivity Questionnaire (A-RSQ; [25, 38, 39], a revised version of the Rejection Sensitivity Questionnaire [6]. Participants were presented with vignettes of nine different situations in which rejection might be possible (e.g. “After a bitter argument, you call or approach your significant other because you want to make up.”). Two items with a 6-point response scale were used to assess participants’ perceptions of each situation: (1) the likelihood of rejection (1 = *very unlikely*; 6 = *very likely*); (2) the degree of concern regarding the possible outcome of each situation (1 = *unconcerned*; 6 = *very concerned*). Following the recommendations of the original measure, we multiplied the concern ratings by the expectancy ratings for each situation and averaged the scores. The internal consistency was high (α *=* 0.81) for the concern scale and moderate for the expectancy scale (α *=* 0.71).

*Affect.* At each of the 28 reports, participants were asked how they felt “in the moment” and had to answer the question by reporting on four different descriptors of negative affective states: “irritated”, “lonely”, “depressed”, and “worried”. For each descriptor, participants reported how they felt on a 10-point scale (1 = *not at all*; 10 = *very*). The four negative item reports were averaged to obtain a negative affect score. Using McDonald’s omega, the internal consistency for negative affect was satisfactory for both the women (within: ω = 0.67; between: ω = 0.86) and the men (within: ω = 0.68; between: ω = 0.89).

#### Perceptions of negative interactions

To build a variable that reflected perceptions of negative interpersonal interactions and disregard from the partner, we used momentary reports on a variety of interpersonal experiences. Specifically, we chose assessments of situations that have been used in previous literature as indicators of potential rejection from a significant other [6, 31, 40]. Two questions directly assessed whether participants felt [1] rejected and [2] mistreated by their partner in the last hour, using a 10-point scale (1 = *not at all*; 10 = *very*). We also included another variable that reflected the perception of the partner as being distant in the relationship (“During our last contact, my partner was aloof.”). We then computed a mean of the three items; this represented perceptions of negative interactions with the partner. Higher scores indicated more negative perceptions of negative interactions. Internal consistency was satisfactory for women (within: ω = 0.68; between: ω = 0.89) and men (within: ω = 0.73; between: ω = 0.96). Overall, out of all the participant’s daily reports, 23.6% were reports of perceptions of negative interpersonal interactions; this represented 1892 reports out of a potential total of 6128 reports. We centred this variable at the person mean to reflect within-person variability in perceptions of negative interactions.

#### Relationship satisfaction

Participants rated their satisfaction with their relationship each day with a single item (“At the moment, I feel satisfied in my relationship.”) on a 10-point scale (1 = *not at all*; 10 = *extremely*). Higher scores indicated higher satisfaction with the relationship. We computed a single score per person, averaged across all ratings, and centred this variable at the grand mean. We tested this variable as a control variable alongside rejection sensitivity.

### Analyses

The current study included dyadic data that featured repeated measures. We used a multilevel modelling approach to model the non-independence of emotional states at the within- and between-person levels [41], testing equations that included separate coefficients for the two partners in the couple. Because our sample included mixed-gender couples, dyad members were distinguishable by their reported gender [42]. Daily reports of both partners (Level 1) were modelled as nested within couples (Level 2), while the women and men partners were represented by separate coefficients in the equation. The effects of perceptions of negative interactions (Level 1 predictor) were estimated at the within-couple level. At this level, we only examined actor effects (H1 and H2), with participants’ own reported perceptions of negative interactions predicting their ratings of negative emotions at the same time points (for H1) and at two subsequent time points (for H2). The effects of rejection sensitivity (Level 2 predictor) were modelled at the between-person level, along with the control variables (i.e. average negative emotions and relationship satisfaction). For H3a and H3b, we also examined partner effects, testing whether the participants’ rejection sensitivity predicted their partners’ ratings of negative emotions. All the variables entered at Level 2 were centred at the grand mean.

H1 was tested based on Eq. 1 (for clarity, Eq. 1 does not display the parameters for relationship satisfaction, which we incorporated alongside rejection sensitivity):

1 $$\displaylines{ NEGATIVE\,EMOTION{S_{ij}} = {b_{0j}} + {b_{1j}}(PERCEPTIONS\,OF\, \cr NEGATIVE\,INTERACTION{S_{tj}}) \cr + \,{b_2}\left( {{\text{REJECTION SENSITIVIT}}{{\text{Y}}_j}} \right) \cr + \,{b_{3j}}({\text{REJECTION SENSITIVIT}}{{\text{Y}}_j}* \cr PERCEPTIONS\,OF\,NEGATIVE \cr INTERACTION{S_{tj}})\, + \,{b_{4j}}(PRIOR \cr NEGATIVE\,EMOTIO{N_{tj}}) + \,{r_{tj}} \cr}$$

*Negative emotions*_*tj*_ represent the current report of negative emotions from one participant *i* (man or woman) at time *t*. The estimate *b*_*0j*_ reflects the mean level of a participant’s report of negative emotions when all other predictors are held constant. The estimate for *b*_*1j*_ reflects the within-subject actor effect of perceptions of a participant *i* at time *t*; that is, the association between the perceived negative interactions of a person with their own negative emotions. The estimate *b*_*2j*_ reflects the between-subject actor effect of rejection sensitivity of participant *i.* The estimate for *b*_*3j*_ reflects the interaction effect of a participant’s rejection sensitivity and their own perception of negative interactions at time *t*. This estimate represents a cross-level interaction and can be interpreted as the moderator effect of rejection sensitivity on the effect of participant’s perceived rejection on their negative emotions. The estimate *b*_*4j*_ captures the extent to which the current emotion report is predicted by the prior emotion report (the autocorrelation). Controlling for the prior emotion report renders the outcome interpretable as a change score. The error term *r*_*tj*_ reflects the residual variance. For the H1, H3a and H3b models, we estimated random intercepts and random slopes. For H2, because there was no convergence, we did not estimate random slopes.

To test whether negative emotional states decreased more slowly in rejection sensitive individuals than in their less rejection sensitive counterparts (H2), we examined whether rejection sensitivity was associated with negative emotion trends in the hours after the perception of a negative interaction. To this end, we analysed data from the time points where negative interactions were reported as well as the two subsequent reports. This allowed us to estimate a linear slope that reflected a linear negative emotion trend over three time points at Level 1, starting at the time of perceived negative interaction. These trends were estimated using a trend variable that was coded to indicate the temporal sequence of reports after the negative interaction perception (0 = negative interaction; 1 and 2 for the two subsequent reports, respectively). In this model, the intercept captured the negative affect at the report of a perception of a negative interaction, and the estimate for the trend variable captured the linear trend of negative emotional states after the perception of a negative interaction. Finally, we added the interaction term between the participant’s rejection sensitivity score and the trend variable. The estimate for the interaction term reflected the degree to which participants’ degree of linear change in negative emotional states after perceiving rejection differed as a function of their level of rejection sensitivity.

Finally, to test whether dyadic patterns of rejection sensitivity might be predictive of stronger negative emotional states following rejection-related perceptions (H3a, H3b), we extended the models at Level 2 by including the partner’s rejection sensitivity and the interaction term between both partners’ rejection sensitivity scores as well as the participant’s own rejection sensitivity variable. To examine the effect of individual and dyadic rejection sensitivity on emotional responses to and emotional dynamics after perceived rejection, we estimated the effects of the cross-level interaction terms between rejection sensitivity predictors and the perceived rejection parameter (H3a), or the linear time trends (H3b). For all the hypothesis tests, we also controlled for relationship satisfaction. However, the H3b model did not include the interaction terms due to lack of convergence.

To ensure that our results could be discussed in the light of possible gender differences, we tested and compared a model that distinguished between partners against a model that did not distinguish between partners [43]. Because a significantly better fit resulted for the models that distinguished the partners by gender, these are the results we present in this paper (model for H1: *X*^*2*^(25) = 595.89, *p* < .001; model for H2: *X*^*2*^(8) = 215.23, *p* < .001; model for H3a: *X*^*2*^(16) = 649.51, *p* < .001; model for H3b: *X*^*2*^(15) = 255.10, *p* < .001). The models were run in R using the nlme [44, 45]. The datasets generated and analysed in the current study are available in the Open Science Framework (OSF) repository, https://osf.io/az9vg/3.

## Results

### Descriptive statistics

The correlation matrix for the within and between subjects is presented in Table 1. The average rejection sensitivity scores were comparable for men (*M* = 5.80; *SD* = 2.78) and women (*M* = 5.77; *SD* = 3.23) and the difference was not significant (paired-samples *t*(141) = 0.07, *p* = .942). The mean level of perceptions of negative interactions was *M* = 0.67 (*SD* = 0.92) for men and *M* = 0.41 (*SD* = 0.86) for women, and the gender difference was significant (paired-samples *t*(143) = 2.86, *p* = .005). Men and women reported slightly different levels of negative affect (men: *M* = 1.03; *SD* = 0.92; women: *M* = 0.85; *SD* = 0.90; paired-samples *t*(143) = 1.89, *p* = .061). On average, both men and women reported high levels of satisfaction with their relationship (men: *M* = 9.07; *SD* = 1.08; women: *M* = 9.15; *SD* = 0.96) and there were no significant differences between the two partners (paired-samples *t*(143) = –0.95; *p* = .345).

**Table 1:** Correlation matrix for between and within subjects

	1	2	3	4	5	6	7	8
1. Negative affect W								
2. Perceptions of negative interactions W	0.78**							
3. Rejection sensitivity W	0.13	0.11						
4. Relationship satisfaction W	–0.32**	–0.39**	–0.23**					
5. Negative affect M	0.25**	0.25**	0.08	–0.34**				
6. Perceptions of negative interactions M	0.21*	0.30**	0.09	–0.44**	0.68**			
7. Rejection sensitivity M	0.11	0.08	0.09	–0.18*	0.34**	0.42**		
8. Relationship satisfaction M	–0.17*	–0.28**	–0.19*	0.57**	–0.53**	–0.71**	–0.29**	

*Note* W = Women; M = Men; * *p* < .05. ** *p* < .01

### Association of higher rejection sensitivity with stronger negative affect

For H1, we examined whether individuals higher in rejection sensitivity experienced higher negative emotions when perceiving negative interactions, compared to less rejection sensitive individuals. As shown in Table 2, when perceiving negative interactions, we found a significant increase in both men’s and women’s negative affect (men: *b* = 0.193, *p* < .001; women: *b* = 0.358, *p* < .001). However, when reporting perceptions of negative interactions, rejection sensitivity in both men and women was not associated with changes in negative emotional states since the prior report of affect (men: *b* = –0.001, *p* = .984; women: *b* = –0.026, *p* = .125). Controlling for relationship satisfaction, higher relationship satisfaction predicted lower negative emotions for both women (*b* = –0.213; *p* < .001) and men (*b* = –0.451; *p* < .001). Moreover, when reporting perceptions of negative interactions, men with higher levels of relationship satisfaction reported lower levels of negative emotions (*b* = –0.062; *p* = .010), but this was not the case for women (*b* = –0.061; *p* = .195).

**Table 2:** Momentary associations for H1: perceptions of negative interactions, rejection sensitivity and negative emotions

	Negative emotions				
	Estimate	*SE*	95% CI		*p*
Variable			*LL*	*UL*	
Rejection sensitivity W	0.031	0.016	–0.001	0.062	0.053
Rejection sensitivity M	0.059	0.023	0.013	0.258	0.013*
Relationship satisfaction W	–0.213	0.054	–0.319	–0.107	< 0.001
Relationship satisfaction M	–0.451	0.060	–0.568	–0.334	< 0.001
Perceptions of negative interactions W	0.363	0.040	0.264	0.461	< 0.001
Perceptions of negative interactions M	0.192	0.033	0.127	0.258	< 0.001
Previous negative emotions W	0.082	0.023	0.037	0.127	< 0.001
Previous negative emotions M	0.116	0.024	0.068	0.164	< 0.001
Perceptions of negative interactions W x RS W	–0.023	0.016	–0.054	0.008	0.153
Perceptions of negative interactions M x RS M	0.001	0.012	–0.022	0.024	0.941
Relationship satisfaction W x Perceptions of negative interactions W	–0.061	0.047	–0.153	0.031	0.195
Relationship satisfaction M x Perceptions of negative interactions M	–0.062	0.024	–0.110	–0.015	0.010*
Previous negative emotions W x Perceptions of negative interactions W	0.033	0.018	–0.002	0.067	0.062
Previous negative emotions M x Perceptions of negative interactions M	–0.008	0.017	–0.041	0.026	0.663

Note ***p < .001, **p < .01, *p < .05. W = Women; M = Men; RS = Rejection sensitivity

In H2, we expected that following perceptions of negative interactions from the partner, high rejection sensitive individuals would show a slower decrease in negative emotions than their less rejection sensitive counterparts. The results are presented in Table 3. Rejection sensitivity was not associated with the degree to which individuals recovered from their negative emotions after perceiving negative interactions (men: *b* = –0.026, *p* = .159; women: *b* = 0.006, *p* = .685). Moreover, for men, the recovery of negative emotion after perceiving negative interactions was dependent on relationship satisfaction (*b* = –0.112, *p* = .009).

**Table 3:** Momentary associations for H2: rejection sensitivity and emotional recovery of negative emotions after perceptions of negative interactions from the partner

	Negative emotions				
Variable	Estimate	*SE*	95% CI		*p*
			*LL*	*UL*	
Rejection sensitivity W	0.042	0.025	–0.008	0.091	0.100
Rejection sensitivity M	0.050	0.029	–0.007	0.106	0.085
Recovery W	–0.269	0.044	–0.355	–0.183	< 0.001
Recovery M	–0.165	0.044	–0.252	–0.079	< 0.001
RelSat W	–0.223	0.079	–0.377	–0.069	0.005**
RelSat M	–0.405	0.071	–0.543	–0.267	< 0.001
Recovery W x RS W	0.006	0.014	–0.022	0.034	0.685
Recovery M x RS M	–0.026	0.016	–0.058	0.005	0.106
Recovery W x RelSat W	0.065	0.045	–0.025	0.153	0.159
Recovery M x RelSat M	–0.112	0.043	–0.200	–0.028	0.009**

Note ***p < .001; **p < .01; * *p* < .05. W = Women; M = Men; RS = Rejection sensitivity; RelSat = Relationship satisfaction

### Dyadic patterns of rejection sensitivity and modulation of negative affect

To examine H3a, we tested whether dyadic patterns of rejection sensitivity were associated with stronger negative emotions after perceptions of negative interactions (see Table 4). Specifically, we expected that mutually high rejection sensitivity would be associated with more negative affect in partners compared to when only one or none of the partners scored relatively high in rejection sensitivity. We propose that the three-way interaction between both partners’ rejection sensitivity and perceptions of negative interactions captures the extent to which the dyadic rejection sensitivity scores explain the additional variance in perceptions of negative interactions that predict negative emotions, over and above the partners’ own rejection sensitivity scores. Testing this interaction indicated that the combination of partners’ rejection sensitivity did not explain the between-person differences in negative emotional states when reporting perceptions of negative interactions (men: *b* = –0.001, *p* = .965; women: *b* = 0.001, *p* = .947).

Next, we examined whether negative emotion trends after perceptions of negative interactions were associated with dyadic rejection sensitivity patterns (H3b). The results are presented in Table 5. We propose that the interaction between both partners’ rejection sensitivity scores and recovery captures the effects of the dyadic combination of rejection sensitivity scores and emotional recovery after reports of negative interactions, beyond individual rejection sensitivity scores. Testing this did not confirm our expectations; dyadic patterns of rejection sensitivity did not explain the variance in trends of negative emotional states after perceptions of negative interactions for women (*b* = –0.006, *p* = .327) or men (*b* = –0.003, *p* = .622). That is, both rejection sensitive individuals and their rejection sensitive partners did not show a slower recovery of negative emotions after perceiving rejection.

**Table 4:** Momentary associations for H3a: dyadic patterns of rejection sensitivity predicting negative emotions when perceiving negative interactions

	Negative emotions				
Variable	Estimate	*SE*	95% CI		*p*
			*LL*	*UL*	
Rejection sensitivity W	0.028	0.019	–0.009	0.065	0.142
Rejection sensitivity M	0.064	0.025	0.016	0.112	0.010
Perceptions of negative interactions W	0.391	0.050	0.293	0.489	< 0.001
Perceptions of negative interactions M	0.221	0.034	0.154	0.289	< 0.001
RS W partner	0.021	0.022	–0.022	0.65	0.335
RS M partner	–0.004	0.020	–0.044	0.035	0.834
RS W x Perceptions of negative interactions W	–0.006	0.016	–0.037	0.025	0.702
RS M x Perceptions of negative interactions M	0.003	0.012	–0.021	0.028	0.787
RS W x RS W partner	0.001	0.007	–0.013	0.014	0.982
RS M x RS M partner	–0.008	0.008	–0.024	0.007	0.284
RS W partner * Perceptions of negative interactions W	0.004	0.020	–0.037	0.042	0.859
RS M partner * Perceptions of negative interactions M	0.008	0.011	–0.014	0.031	0.459
RS W x RS W partner x Perceptions of negative interactions W	0.002	0.006	–0.010	0.014	0.757
RS M x RS M partner x Perceptions of negative interactions M	0.001	0.004	–0.008	0.009	0.891
Relationship satisfaction W	–0.239	0.064	–0.364	–0.115	< 0.001
Relationship satisfaction M	–0.408	0.062	–0.530	–0.286	< 0.001

Note ***p < .001, **p < .01, *p < .05. W = Women; M = Men; RS = Rejection sensitivity

**Table 5:** Momentary associations for H3b: dyadic patterns of rejection sensitivity on negative affect recovery after perceptions of negative interactions

	Negative emotions				
Variable	Estimate	*SE*	95% CI		*p*
			*LL*	*UL*	
Rejection sensitivity W	0.036	0.025	–0.013	0.085	0.152
Rejection sensitivity M	0.049	0.030	–0.010	0.109	0.106
RS W partner	0.027	0.028	–0.027	0.082	0.327
RS M partner	–0.005	0.025	–0.054	0.043	0.831
RS W x RS W partner	0.004	0.009	–0.014	0.021	0.684
RS M x RS M partner	–0.010	0.010	–0.032	0.010	0.335
RS W x recovery W	0.001	0.015	–0.029	0.031	0.959
RS M x recovery M	–0.018	0.017	–0.052	0.016	0.292
RS W partner x recovery W	–0.005	0.018	–0.039	0.030	0.787
RS M partner x recovery M	–0.003	0.015	–0.032	0.026	0.842
RS W x RS W partner x recovery W	–0.006	0.006	–0.017	0.005	0.291
RS M x RS M partner x recovery M	–0.002	0.006	–0.014	0.009	0.672
Relationship satisfaction W	–0.237	0.077	–0.388	–0.086	0.002
Relationship satisfaction M	–0.389	0.073	–0.531	–0.245	< 0.001

Note ***p < .001, **p < .01, *p < .05. W = women; M = men; RS = rejection sensitivity

## Discussion

The current study aimed to investigate whether individuals’ rejection sensitivity and dyadic patterns of rejection sensitivity were associated with higher and persistent emotional responses in the context of daily perceptions of negative interactions. H1 predicted that when perceiving negative interactions, individuals with higher levels of rejection sensitivity would report more negative affect than their less rejection sensitive counterparts. The results did not support this hypothesis for either men or women. First, the data showed a strong positive association between perceptions of negative interactions and elevated negative emotional states, suggesting that individuals reported stronger negative emotional states when they reported perceptions of negative interactions compared to when they reported no perceptions of negative interactions, which is in line with the results of previous studies [18, 21]. However, the results suggest that individual differences in the strength of this effect were not attributable to differential levels of rejection sensitivity. In other words, individuals with higher levels of rejection sensitivity did not report stronger negative emotions when they perceived rejection from their partner. Moreover, H2 predicted that following perceptions of negative interactions, rejection sensitive individuals would recover less rapidly from these perceptions, as reflected in a slower decrease in negative emotions. The data did not support this prediction; rejection sensitive individuals did not differ significantly in their emotional recovery compared to less rejection sensitive individuals.

There are several possible reasons for the absence of rejection sensitivity effects on emotional responses and recovery. First, the items used to reflect perceptions of negative interactions might not point to situations that are challenging enough to reveal rejection sensitivity effects. The literature suggests that individuals may be sensitive to rejection in specific situations. For instance, men tend to perceive rejection in conflictual situations or in situations that threaten their status [3] whereas for women, perceptions of negative interactions occur when they perceive their partner to be inattentive [46]. Moreover, rejection sensitivity in men has been associated with heightened jealousy and controlling behaviours after rejection [6] and aggression [46, 47]. Rejection sensitive women are less supportive, distance themselves and tend to conform to maintain their relationship [6, 27, 40]. These contrasting differences in men’s and women’s perceptions of rejection and their responses to them may be reflected in our results. Indeed, the questionnaire items we used reflected subjective perceptions of negative interactions and were quite broad to specifically capture an interaction where rejection might have occurred. In our sample, the emotions of both high rejection sensitive men and women were not affected by their perceptions of negative interactions. It may be that the questionnaire items did not represent threatening interactions that were sufficient to trigger stronger negative emotions. Alternatively, the participants might not have felt prompted to respond in self-protective ways or to seek closeness with their partner [4], and this may have been reflected in the absence of stronger emotional responses and slower downregulation.

It is also possible that rejection sensitive individuals respond to perceptions of threatening interactions by attempting to reduce emotional arousal, thus suppressing their emotions to avoid being hurt and to protect themselves and their relationship [25, 40]. However, by engaging in such behaviours and choosing distance over closeness [4], such individuals may not benefit from their partner’s help in regulating their negative emotions [1]. In particular, if individuals feel chronically undervalued [7], these patterns may regularly repeat and accumulate. In addition, similarly to individuals with avoidance attachment orientation [20, 48], through interactions with their partner, a rejection sensitive person may have learned to divert their attention away from such threats. As a result, they may not show or report distress when faced with potential rejection [25].

H3a and H3b predicted dyadic effects of rejection sensitivity, expecting that when both partners are highly rejection sensitive, they experience stronger negative emotions compared to when only one or none of the partners have higher levels of rejection sensitivity. We also expected that when both partners were rejection sensitive, they would remain longer in a negative emotional state related to rejection. This assumption was based on the reasoning that mutual sensitivity to rejection enhances negative reciprocal dynamics between partners, and thus increases response intensity and prolongs negative emotional states. However, our results showed that neither women nor men with higher levels of rejection sensitivity reported stronger negative emotional responses to perceived negative interactions when their partner was also rejection sensitive. Furthermore, neither men nor women experienced prolonged emotional states compared to when one or none of the partners were highly rejection sensitive.

Our data did not show negative patterns of reciprocity between rejection sensitive partners. Research on insecure attachment, a correlate of rejection sensitivity [6], offers a possible explanation for this finding [29], suggesting that anxious-avoidant and anxious-ambivalent individuals are more likely to be rejection sensitive [49]. When faced with rejection from a significant other, individuals with an avoidant attachment style inhibit strong emotions to avoid threatening thoughts that might activate their attachment needs. Such individuals are also less likely to react with anger when they are confronted with their partner’s negative behaviour and are less distressed after a hurtful event [20]. Instead, they typically distance themselves from their partner and show more hostility [29]. Furthermore, evidence suggests that when both partners are insecure, they engage in mutual avoidance and withdraw from communication [35]. To the extent that these findings can be applied to rejection sensitivity, mutual use of avoidance strategies in couples with higher levels of rejection sensitivity in both partners could feed avoidance cycles. However, these negative dyadic dynamics are more likely to manifest in heightened avoidance [25] than in intense mutual expressions of negative emotions. If this effect was active in our study, high rejection sensitive individuals may not have reported stronger negative emotions when they perceived negative interactions. Nonetheless, they may have felt more distant, and these dynamics will still contribute to dysfunctional interactions in the long run [50]. Another possibility is that these individuals may have attempted to de-escalate negative interactions, rather than engage in emotional avoidance or disengagement. It is important to stress that these possibilities are all very speculative and that further research is needed to confirm whether they are viable explanations.

Finally, when controlling for relationship satisfaction, our results showed that higher levels of relationship satisfaction in men predicted lower negative emotions, and a slower recovery from the perceived negative interactions. We did not expect these results and offer some possible explanations for them. Interestingly, while high levels of satisfaction in men predicted lower negative emotions, men recovered less quickly from these negative emotional states. A reason for these results may be that individuals highly satisfied in their relationship hold high expectations of their romantic relationship and their partner [51]. As a result, these individuals may still report negative emotion, though significantly less, but it may take them longer to recover from this emotional state. They may react with a heightened sensitivity to such interactions because they consider and care for their romantic relationship more [51]. Besides, the context may also contribute to such responses. The transition to parenthood is often depicted as a joyful period, where future parents rejoice in this common challenge [52]. Thus, partners may report higher levels of satisfaction, which would render them more sensitive to threatening interactions. In addition, disclosing emotions contribute to relationship maintenance [53], which suggest that reporting negative emotions does not point to negative relational processes. Instead, partners may need such moments to increase intimacy and to foster their relationship [51]. Additional research specifically focusing on these aspects is needed to understand the mechanisms that underlie them. How individuals respond to their partner, and their ability to regulate and recover from their emotions, plays a crucial role in relationship satisfaction and how couples deal with conflict, for example [54].

The current study has several limitations. First, high levels of rejection sensitivity were relatively rare in our sample. Therefore, most participants may be unlikely to display the strong emotional responses to negative interactions needed to reveal higher levels of rejection sensitivity. Second, the measure of rejection sensitivity was based on self-reported experiences in response to general interpersonal situations with different people. Such reports do not necessarily reflect rejection responses to everyday interactions, or in this context, to a romantic partner. Self-reports on specific daily rejection-relevant interactions with the partner may capture different types of responses that represent more immediate reactions to rejection. Moreover, the questions included in the momentary assessment did not all refer to the same time points and this may have been a source of systematic error variance. Importantly, when measuring emotions after a certain event, a three- to six-hour difference between each report might have been too wide to capture relevant emotions. Hence, further studies including a smaller time difference between each self-report questionnaire are needed to fully grasp the potential effects of rejection on emotional reactions; this would allow a more fine-grained measurement (e.g. in minutes rather than hours). Similarly, although we were primarily interested in emotion dynamics as an outcome, the distinction between soft affect (e.g. hurt, sadness) and hard affect (e.g. anger) in relation to rejection may be of interest for future research, as these affective responses may serve different social functions relevant to interpersonal interactions [55]. Whereas soft affects may reflect vulnerability and promote or facilitate affiliation, hard affects are associated with assertiveness and threat and tend to promote interpersonal distance [56, 57].

Part of the present study examined reports of emotions and perceptions of negative interactions at the same point in time. While some useful data was obtained, this design precluded obtaining causal effects. Therefore, future studies may aim at establishing predictions of prospective change or use experimental approaches to allow for stronger causal interpretations. Finally, the couples in our sample were all expecting their first child. This situation is unique and the relationship experience of expectant couples may differ from that of other types of couples. Hence, the generalizability of our results is limited. It is unclear how this unique relationship situation may have affected our results. On the one hand, emotional responses may be different at this time. Partners may be more focused on their future child, and be more willing to override negative relational sentiments through experiences of togetherness and cooperation [58–60]. On the other hand, although expectant couples continue to experience common difficulties in their daily interactions [61–64], the period of expecting a first child is associated with adjustment difficulties and increased stress [65, 66]. This may exacerbate relationship insecurities [67] and thus, negatively affect perceptions of the relationship [68]. Moreover, rejection sensitive individuals in committed long-term relationships may show different emotional patterns as a response to rejection compared to individuals in the early stages of a relationship, as they may have developed regulation strategies to manage their relational experiences [69].

While our results did not show an effect of rejection sensitivity on the emotion regulation capacities of individuals, therapists may nonetheless benefit from these findings. Rejection sensitive individuals are concerned about possible rejection; therefore, it might be of interest to specifically target relationship-based anxiety. For instance, a brief psychoeducational intervention has shown how behaviours such as self-silencing or partner accommodation can change significantly following intervention [70]. Moreover, emotionally focused couple therapy is associated with decreased anxious and avoidance attachment [71]. In both options, partners learn how to communicate with each other [70] and de-escalate negative interactions [71]. Such interventions might prevent the perpetuation of negative feedback cycles through both partners’ dyadic communication and understanding of each other’s experiences, especially when they are both highly sensitive to rejection.

In conclusion, the current study did not fully support the notion that rejection sensitivity plays a role in modulating emotional responses and regulation following perceptions of negative interactions in intimate relationships, or that dyadic patterns of rejection modify emotional responses on a daily basis. Rejection sensitive individuals may suppress their emotions and protect themselves over their relationship, which in the long run, may be detrimental to the relationship. Additionally, the absence of emotional responses underscores the need to identify what kind of interactions may prompt negative emotions in rejection sensitive individuals. Finally, the identified association between relationship satisfaction and negative emotional responses is a new finding and a possible avenue for future research on emotion regulation and relationship outcomes.

Our findings are important because they contribute to the gap in the research on daily emotional dynamics and rejection sensitivity. Rejection sensitivity emotional responses may not necessarily become more apparent in the specific rejection interactions perceptions we used. Future studies should shed more light on the relevance of rejection sensitivity for negative emotion dynamics in such relationships by focusing on samples with higher levels of rejection sensitivity and adapting diary studies to more accurately capture the possible aftermath of perceptions of negative interactions.

## Data Availability

The datasets analyzed in this study are available on the OSF repository, https://osf.io/az9vg/.

## Footnotes

1 Contrary to the preregistration protocol, because of a lack of coherence, we did not include Hypothesis 2b, which predicted a slower recovery of positive emotions in rejection sensitive individuals, in the current paper. The results can be found online: https://osf.io/gtj5u/.

2 Contrary to the preregistration protocol for the current study, we did not test our initial Hypothesis 3b, in which we predicted more frequent negative emotions when both partners are rejection sensitive. Because the available data on emotions were not categorical, we could not straightforwardly assess the frequency of negative emotions. Therefore, we did not include this hypothesis in the paper.

3 Several changes have occurred since the preregistration, partly due to the operationalization of our hypotheses as well as to addressing reviewers’ comments. Mainly, measures of rejection sensitivity, perceptions of negative interactions, and the time trend variable were modified to better respond to our hypotheses.
